# Distinct External Signals Trigger Sequential Release of Apical Organelles during Erythrocyte Invasion by Malaria Parasites

**DOI:** 10.1371/journal.ppat.1000746

**Published:** 2010-02-05

**Authors:** Shailja Singh, M. Mahmood Alam, Ipsita Pal-Bhowmick, Joseph A. Brzostowski, Chetan E. Chitnis

**Affiliations:** 1 Malaria Group, International Centre for Genetic Engineering and Biotechnology (ICGEB), New Delhi, India; 2 Laboratory of Malaria and Vector Research (LMVR), National Institute of Allergy and Infectious Diseases (NIAID), National Institutes of Health (NIH), Rockville, Maryland, United States of America; 3 Laboratory of Immunogenetics Imaging Facility, National Institute of Allergy and Infectious Diseases (NIAID), National Institutes of Health (NIH), Rockville, Maryland, United States of America; National Institute for Medical Research, United Kingdom

## Abstract

The invasion of erythrocytes by Plasmodium merozoites requires specific interactions between host receptors and parasite ligands. Parasite proteins that bind erythrocyte receptors during invasion are localized in apical organelles called micronemes and rhoptries. The regulated secretion of microneme and rhoptry proteins to the merozoite surface to enable receptor binding is a critical step in the invasion process. The sequence of these secretion events and the external signals that trigger release are not known. We have used time-lapse video microscopy to study changes in intracellular calcium levels in *Plasmodium falciparum* merozoites during erythrocyte invasion. In addition, we have developed flow cytometry based methods to measure relative levels of cytosolic calcium and study surface expression of apical organelle proteins in *P. falciparum* merozoites in response to different external signals. We demonstrate that exposure of *P. falciparum* merozoites to low potassium ion concentrations as found in blood plasma leads to a rise in cytosolic calcium levels through a phospholipase C mediated pathway. Rise in cytosolic calcium triggers secretion of microneme proteins such as the 175 kD erythrocyte binding antigen (EBA175) and apical membrane antigen-1 (AMA-1) to the merozoite surface. Subsequently, interaction of EBA175 with glycophorin A (glyA), its receptor on erythrocytes, restores basal cytosolic calcium levels and triggers release of rhoptry proteins. Our results identify for the first time the external signals responsible for the sequential release of microneme and rhoptry proteins during erythrocyte invasion and provide a starting point for the dissection of signal transduction pathways involved in regulated exocytosis of these key apical organelles. Signaling pathway components involved in apical organelle discharge may serve as novel targets for drug development since inhibition of microneme and rhoptry secretion can block invasion and limit blood-stage parasite growth.

## Author Summary

Malaria remains a major public health problem in many parts of the tropical world. All the clinical symptoms of malaria are attributed to the blood stage of the parasite life cycle during which Plasmodium merozoites invade and multiply within host erythrocytes. Invasion by Plasmodium merozoites is a complex process that requires multiple molecular interactions between the invading parasite and target erythrocyte. Parasite proteins that mediate such interactions are localized in membrane bound internal organelles at the apical end of merozoites called micronemes and rhoptries. The timely secretion of microneme and rhoptry proteins to the merozoite surface to allow receptor binding is a crucial step in the invasion process. In this study, we demonstrate that exposure of *Plasmodium falciparum* merozoites to low potassium ion concentrations as found in blood plasma provides the natural signal that triggers a rise in intracellular calcium, which in turn triggers secretion of microneme proteins to the merozoite surface. Subsequently, binding of released microneme proteins with erythrocyte receptors provides the signal for release of rhoptry proteins. These studies open the path for analysis of signal transduction pathways involved in apical organelle secretion. A clear understanding of these pathways will enable development of inhibitors that block secretion of key parasite proteins required for receptor-binding. Such inhibitors will block erythrocyte invasion and inhibit parasite growth, providing promising leads for development of novel drugs against malaria.

## Introduction

Malaria continues to be a major public health problem in tropical regions of the world. It is responsible for significant morbidity and mortality with around 300 to 500 million malaria cases reported annually that result in about 2 million malaria-related deaths [1]. Of the Plasmodium species responsible for human malaria, *Plasmodium falciparum* is the most virulent and accounts for the vast majority of deaths attributed to malaria. Given the rapid spread of drug resistant malaria parasites, there is an urgent need to develop novel intervention strategies including new drugs and effective vaccines to combat malaria.

All the clinical symptoms of malaria are attributed to the blood stage of the parasite life cycle during which Plasmodium merozoites invade and multiply within host erythrocytes. Invasion of erythrocytes by Plasmodium merozoites is a complex multi-step process that is mediated by specific molecular interactions between host receptors and parasite ligands [2]. A clear understanding of the molecular mechanisms involved in erythrocyte invasion could lead to the development of novel approaches to inhibit invasion, limit blood-stage parasite growth and protect against malaria.

Plasmodium species belong to the phylum Apicomplexa and are characterized by the presence of apical membrane bound organelles called micronemes and rhoptries that play important roles in host cell invasion. A number of key parasite ligands that mediate critical interactions with host receptors during invasion are localized in these apical organelles [2]. Studies of the invasion process by light and electron microscopy suggest that the early steps of invasion include merozoite attachment to the erythrocyte surface, apical re-orientation, release of apical organelles including micronemes and rhoptries and formation of an irreversible ‘junction’ between the apical end of the invading merozoite and the target erythrocyte [3]–[6]. The precise timing and sequence of microneme and rhoptry secretion during invasion is not known. Moreover, the external signals and signaling pathways that trigger the coordinated release of microneme and rhoptry proteins remain to be identified.

In case of Plasmodium sporozoites cAMP as well as cytosolic Ca^+2^ have been shown to be involved in signaling mechanisms that trigger microneme secretion during hepatocyte invasion [7]. In *P. falciparum* merozoites rise in intracellular calcium has been implicated in phosphorylation of components of the motility machinery of merozoites, which is critical for invasion [8]. In the related Apicomplexan parasite *Toxoplasma gondii*, intracellular calcium regulates a number of critical processes including parasite motility, egress from host cells and secretion of microneme proteins [9]–[12]. Treatment of *T. gondii* tachyzoites with calcium modulating agents such as the ionophore A23187 or ethanol, which elevate cytosolic calcium levels, trigger release of microneme proteins such as MIC2 that play essential roles in host cell invasion [13]–[15]. Endogenous production of the phytohormone abscissic acid in *T. gondii* tachyzoites has been shown to trigger release of calcium from internal stores resulting in secretion of microneme proteins that mediate host cell invasion [16].

The rhoptries are bulbous membrane bound secretory organelles that are located at the apical end of Apicomplexan parasites. Both rhoptry neck proteins (RONs) as well as proteins localized in the rhoptry bulb are secreted during host cell invasion [17],[18]. *T. gondii* rhoptries contain a number of protein kinases, which play an important role as virulence factors and are secreted into the host cell during invasion [19],[20]. The external signals and signaling mechanisms responsible for secretion of rhoptry proteins are not known in any Apicomplexan parasite.

Here, we have investigated the role of intracellular calcium in the regulated secretion of apical organelles in *P. falciparum* merozoites during invasion. We describe the identification of physiologically relevant external signals that trigger the sequential release of microneme and rhoptry proteins. Importantly, we demonstrate that secretion of microneme and rhoptry proteins is triggered by distinct external signals. Exposure of merozoites to low potassium ion concentrations as found in blood plasma appears to provide the external signal that leads to a rise in cytosolic calcium, which triggers translocation of microneme proteins such as the 175 kD erythrocyte binding antigen (EBA175) [21]–[23] and apical membrane antigen-1 (AMA1) [24] to the merozoite surface. Following release to the merozoite surface, engagement of EBA175 with its receptor, glycophorin A (glyA), restores basal cytosolic calcium levels and triggers the release of rhoptry proteins such as CLAG3.1 [25] and PfRH2b [26]. These observations provide a starting point for the analysis of signaling pathways involved in regulated secretion of apical organelles during invasion. A clear understanding of these pathways may provide novel targets for intervention since inhibition of apical organelle release will block invasion and limit blood-stage parasite multiplication.

## Results

### Intracellular calcium levels in *P. falciparum* merozoites during erythrocyte invasion

**Figure 1:**
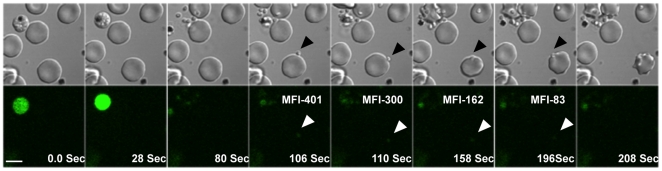
Cytosolic calcium levels in *P. falciparum* merozoites during erythrocyte invasion. Late stage *P. falciparum* schizonts were labeled with Fluo-4AM and added to uninfected erythrocytes to allow re-invasion. Calcium levels were monitored in released merozoites during invasion by time-lapse video microscopy. Both DIC and fluorescence images were acquired at 2 second intervals. Selected pairs of DIC and fluorescence images with time elapsed between frames in seconds (Sec) are shown. The mean fluorescence intensity (MFI) after subtraction of background in an individual merozoite that successfully completed invasion (marked by arrows) is indicated. White bar indicates 5 µm. Also see Supplementary Information, Videos S1, S2 and S3.

Calcium is commonly used as a second messenger in signal transduction pathways that regulate key life processes in eukaryotic cells. Here, we have investigated whether calcium is involved in regulating processes during host cell invasion by *P. falciparum* merozoites. We have used time-lapse video microscopy to study changes in levels of free cytosolic calcium in *P. falciparum* merozoites during erythrocyte invasion. Mature *P. falciparum* schizonts were labeled with the calcium sensitive fluorescence indicator Fluo-4AM and incubated with erythrocytes to allow re-invasion. Free merozoites released from ruptured schizonts were fluorescent indicating presence of high levels of free intracellular calcium (Figure 1 and Videos S1, S2 and S3). Importantly, the distribution of the fluorescence signal observed in Fluo-4AM labeled free merozoites indicates that the calcium indicator disperses uniformly in the merozoite cytoplasm and does not accumulate in any internal organelles (Figure S1). Fluo-4AM can thus be used to report cytosolic calcium concentrations in merozoites. Following interaction with target erythrocytes, the fluorescence intensity in attached merozoites drops prior to invasion indicating a decrease in cytosolic calcium levels (Figures 1 and S2). In contrast, calcium levels remain high in free merozoites that do not attach to erythrocytes over comparable periods of time (Figure S2). These observations indicate that free merozoites released from ruptured schizonts in RPMI medium have high levels of free cytosolic calcium, which drop following attachment with target erythrocytes before invasion proceeds to completion.

### Elevation of intracellular calcium levels in *P. falciparum* merozoites triggers release of microneme proteins but not rhoptry proteins

We wondered whether the elevated cytosolic calcium levels observed in free *P. falciparum* merozoites released from schizonts could trigger the release of apical organelles. We developed methods to isolate viable *P. falciparum* merozoites and study translocation of microneme and rhoptry proteins to the merozoite surface in response to changes in intracellular calcium levels. *P. falciparum* cultures were synchronized by sorbitol treatment and late stage schizonts were allowed to mature and rupture naturally. Released merozoites were separated from unruptured schizonts and uninfected erythrocytes by differential centrifugation. To test their competence for invasion, ∼1×10^6^ merozoites were incubated with ∼1×10^7^ erythrocytes for 18–20 hours to allow invasion and newly invaded rings were scored by Giemsa staining. Based on the invasion rates measured, we estimate that 25.8±3.3% (n = 5) of merozoites invaded erythrocytes successfully to form rings. In parallel experiments, ∼1×10^5^ late stage purified *P. falciparum* schizonts were incubated with ∼1×10^7^ erythrocytes to allow schizont rupture and re-invasion. Newly invaded ring-infected erythrocytes were scored by Giemsa staining. Based on the invasion rates measured and assuming that each ruptured schizont releases ∼10 merozoites, we estimate that 17.4±4.1% of merozoites released naturally following rupture of schizonts invaded erythrocytes successfully. The invasion rates for merozoites isolated as described above and merozoites that invade target erythrocytes directly after release in culture following rupture of mature schizonts are therefore comparable. *P. falciparum* merozoites isolated for use in studies described here are thus viable and retain their competence for invasion.

**Figure 2:**
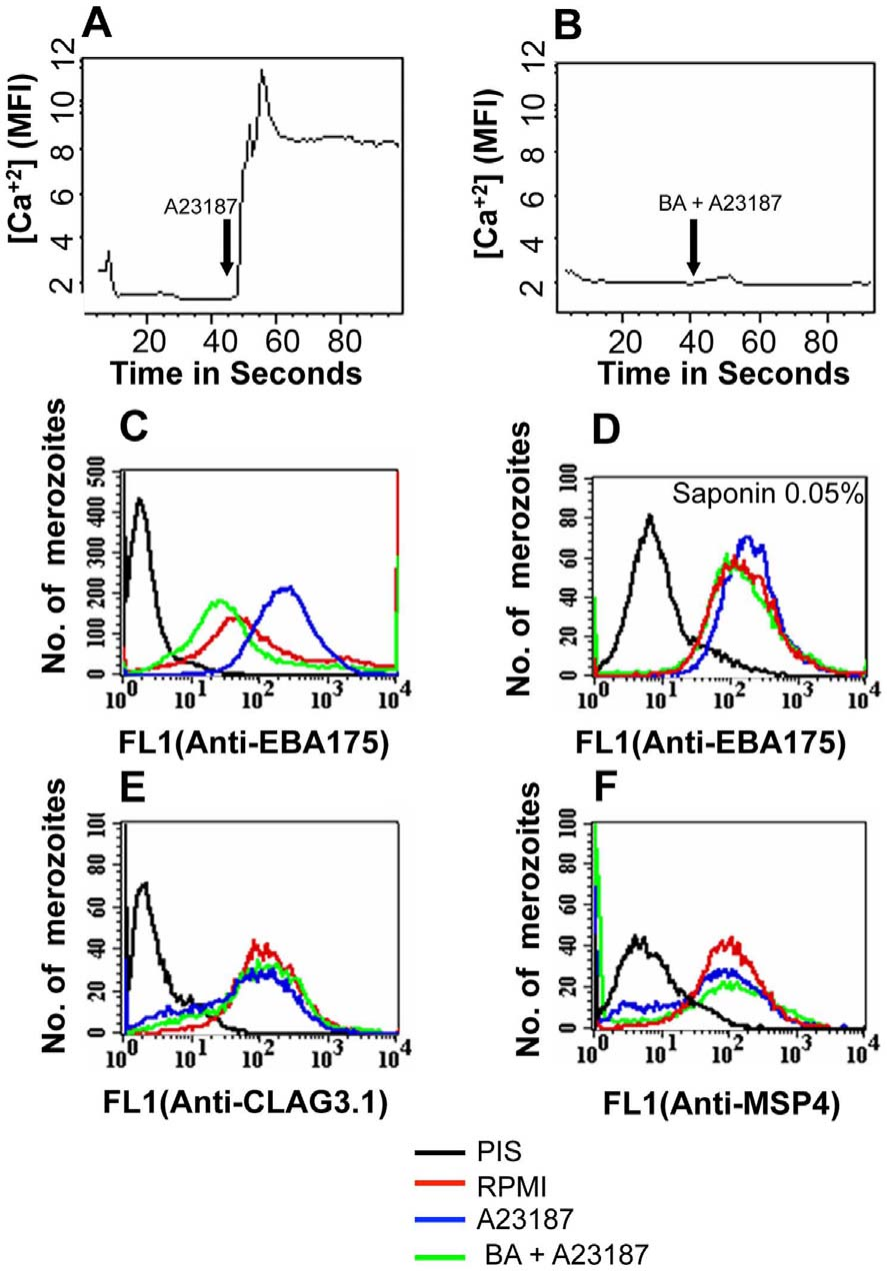
Cytosolic calcium levels and expression of EBA175 on merozoite surface detected by flow cytometry following treatment with calcium ionophore A23187. (**A–B**) *Cytosolic calcium levels in merozoites upon treatment with ionophore A23187*. Changes in cytosolic calcium levels were detected in *P. falciparum* merozoites labeled with Fluo-4AM by flow cytometry after treatment with either A23187 (**A**) or BAPTA-AM followed by A23187 (BA + A23187) (**B**). Mean fluorescence intensities (MFI), which reflect relative levels of free cytosolic calcium [Ca^+2^], measured by flow cytometry are shown as a function of time in seconds. Arrow indicates time-point at which A23187 was added. (**C–F**) *Surface expression of microneme, rhoptry and merozoite surface proteins upon treatment with A23187*. Expression of microneme protein EBA175 (**C**), rhoptry protein CLAG3.1 (**E**) and merozoite surface protein MSP4 (**F**) was detected on surface of *P. falciparum* merozoites isolated in RPMI1640 medium (RPMI, red) or following treatment with either A23187 (A23187, blue) or treatment with BAPTA-AM followed by A23187 (BA + A23187, green) using specific sera by flow cytometry. Microneme protein EBA175 was also detected in merozoites permeabilized with 0.05% saponin using specific sera (**D**). Untreated merozoites stained with pre-immune serum (PIS, black) were used as controls (**C–F**).

**Figure 3:**
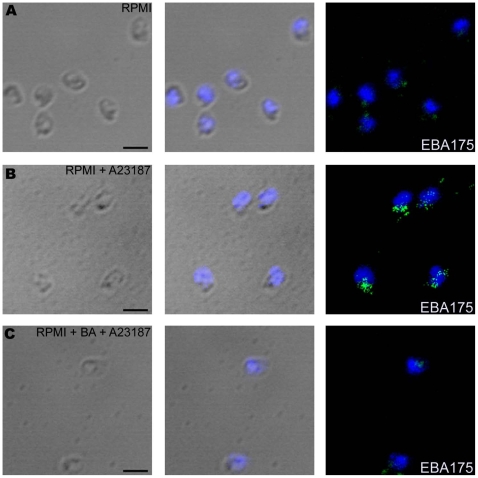
Expression of EBA175 on merozoite surface detected by immunofluorescence assay (IFA) following treatment with calcium ionophore A23187. Expression of microneme protein EBA175 on surface of *P. falciparum* merozoites isolated in RPMI1640 medium (RPMI) (**A**) or following treatment with either A23187 (RPMI + A23187) (**B**) or BAPTA-AM followed by A23187 (RPMI + BA + A23187) (**C**) was detected by IFA using anti-EBA175 rabbit sera followed by FITC-conjugated anti-rabbit IgG goat sera (green). Nuclear DNA was counterstained with DAPI (blue). Bright field, bright field merged with DAPI staining and merged fluorescence images (DAPI and FITC) are shown. Black bar indicates 2 µm.

We developed methods to label isolated *P. falciparum* merozoites with Fluo-4AM and measure cytosolic calcium levels by flow cytometry. Treatment of merozoites with the calcium ionophore A23187 results in an increase in cytosolic calcium (Figure 2A). The rise in free cytosolic calcium is blocked if merozoites are treated with the membrane permeable calcium chelator BAPTA-AM prior to addition of A23187 (Figure 2B). Merozoites isolated in RPMI and incubated with or without A23187 were fixed with p-formaldehyde and stained with specific antisera to detect changes in surface expression of microneme protein EBA175 [27], rhoptry protein encoded by cytoadherence-linked asexual gene 3.1 (CLAG3.1) [25] and merozoite surface protein 4 (MSP4) [28] by flow cytometry. Antiserum raised against a cytoplasmic protein, namely, nucleosome assembly protein-large (NAPL) [29], was used as a control to confirm that only proteins on the merozoite surface are detected by this method. Anti-NAPL serum does not yield any signal with p-formaldehyde-fixed merozoites either by flow cytometry (Figure S3) or by immunofluorescence assay (IFA) (Figure S4). NAPL is detected only if p-formaldehyde-fixed merozoites are permeabilized with saponin (Figure S3 and S4). The fixing method used thus does not permeabilize merozoites and only proteins on the merozoite surface are detected. Surface expression of EBA175 increases upon treatment of merozoites with A23187 (Figure 2C and Table S1). The increase in EBA175 on the merozoite surface is blocked if merozoites are treated with BAPTA-AM prior to treatment with A23187 (Figure 2C and Table S1). The increase in EBA175 levels on the merozoite surface following treatment with A23187 was confirmed by IFA (Figure 3). EBA175 secreted to the merozoite surface in response to the increase in cytosolic calcium is localized at the apical end (Figure 3). Treatment of merozoites with A23187 also triggers the release of microneme protein AMA1 to the merozoite surface (Figure S5 and Table S1). Levels of EBA175 and AMA1 detected in merozoites permeabilized with saponin remain unchanged before and after treatment with A23187 (Figures 2D and S5B). The increase in EBA175 and AMA1 on the merozoite surface following A23187 treatment can thus be attributed to translocation of these proteins to the surface from an intracellular location. Surface expression levels for MSP4, which is constitutively expressed on the merozoite surface, and CLAG3.1, which is localized in rhoptries, remain unchanged after treatment with A23187 (Figure 2E–F and Table S1). These observations indicate that rise in cytosolic calcium specifically triggers translocation of microneme proteins to the merozoite surface.

### Exposure of *P. falciparum* merozoites to low potassium ion concentrations elevates intracellular calcium levels and triggers microneme release

The physiologically relevant external signals that can induce a rise in intracellular calcium during invasion to trigger translocation of microneme proteins to the merozoite surface are not known. Given the drastic differences between the ionic environments in the erythrocyte cytosol and in blood plasma, we wondered whether exposure of merozoites to the ionic environment found in blood plasma could provide the signal for microneme release. The internal ionic environment in erythrocytes is characterized by low [Na^+^] (5 mM), high [K^+^] (140 mM) and low [Ca^+2^] (nanomolar) [30] whereas blood plasma has high [Na^+^] (140 mM), low [K^+^] (5 mM) and high [Ca^+2^] (1 mM).

We used Fluo-4AM to determine whether exposure of merozoites to ionic conditions found in blood plasma can trigger a rise in cytosolic calcium levels. Since Fluo-4AM is not a ratiometric dye, we first measured the *in situ* binding constants for Fluo-4AM in merozoites resuspended in buffers mimicking intracellular (IC buffer – 5 mM NaCl, 140 mM KCl, 1 mM EGTA) and extracellular (EC buffer - 140 mM NaCl, 5 mM KCl, 1 mM CaCl_2_) ionic conditions (Figure S6). The *in situ* binding constants for Fluo-4AM in IC (K_d_ = 1.13 µM) and EC buffers (K_d_ = 1.19 µM) are similar (Figure S6) confirming that Fluo-4AM can accurately report changes in cytosolic calcium concentrations in merozoites under these ionic conditions.

**Figure 4:**
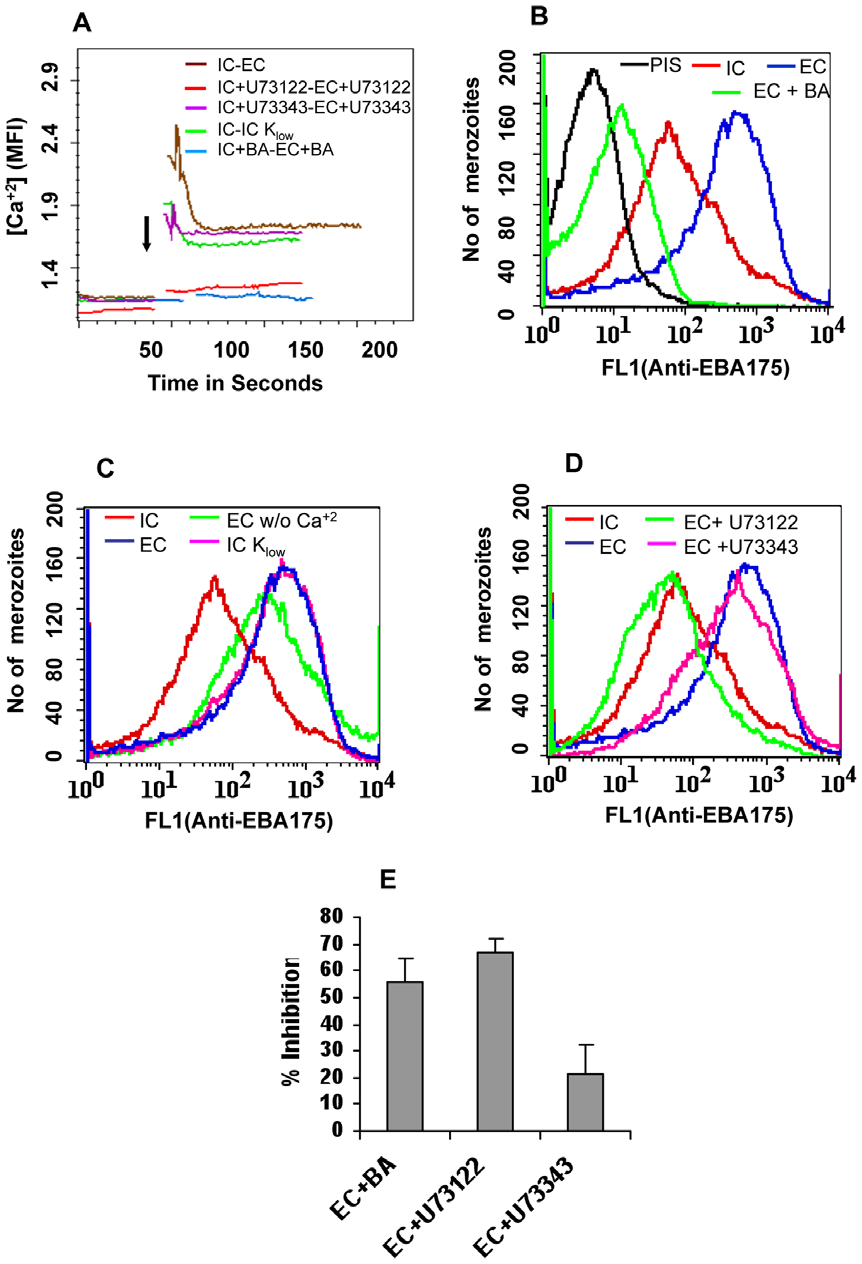
Cytosolic calcium levels and expression of EBA175 on merozoite surface in response to changes in ionic conditions. (**A**) *Cytosolic calcium levels in merozoites under different ionic conditions*. *P. falciparum* merozoites were isolated in buffer mimicking intracellular conditions (IC – 5 mM NaCl, 140 mM KCl, 1 mM EGTA). Merozoites were labeled with Fluo-4AM and cytosolic calcium levels [Ca^+2^] were measured by flow cytometry before and after transfer from IC buffer to buffer mimicking extracellular conditions (EC – 140 mM NaCl, 5 mM KCl, 1 mM CaCl_2_ (IC-EC, brown)) or IC K_low_ buffer (IC K_low_ - 5 mM NaCl, 5 mM KCl, 135 mM choline-Cl, 1 mM EGTA (IC-IC K_low_, green)). Merozoites isolated in IC buffer were transferred to EC buffer after prior treatment with either BAPTA-AM (IC+BA-EC+BA, blue) or U73122 (IC+U73122-EC+U73122, red) or its inactive analog U73343 (IC+U73343-EC+U73343, purple). Mean fluorescence intensities (MFI), which reflect relative levels of free cytosolic calcium measured by flow cytometry, are shown as a function of time in seconds. Arrow indicates time-point at which merozoites were transferred from IC buffer. (**B**) *Surface expression of EBA175 under different ionic conditions*. Expression of EBA-175 was detected on surface of merozoites isolated in IC buffer (IC, red), following transfer from IC to EC buffer (EC, blue) or following transfer from IC to EC buffer after prior treatment with BAPTA-AM (EC + BA, green) using specific sera by flow cytometry. Merozoites isolated in IC buffer and stained with pre-immune serum (PIS, black) were used as controls. (**C**) *Surface expression of EBA175 in response to change in [K^+^]*. Expression of EBA-175 was detected on surface of merozoites isolated in IC buffer (IC, red), after transfer from IC to EC buffer (EC, blue), IC K_low_ buffer (IC K_low_, pink) or EC w/o Ca^+2^ buffer (140 mM NaCl, 5 mM KCl, 1 mM EGTA (EC w/o Ca^+2^, green) using specific sera by flow cytometry. (**D**) *Effect of PLC inhibitor U73122 on surface expression of EBA175*. Expression of EBA175 was detected on surface of merozoites isolated in IC buffer (IC, red), following transfer from IC to EC buffer (EC, blue) or from IC to EC buffer following pre-treatment with either U73122 (EC+U73122, green) or its inactive analog U73343 (EC+U73343, pink) using specific sera by flow cytometry. (**E**) *Invasion rates in presence of calcium signaling pathway inhibitors*. Merozoites were allowed to invade erythrocytes in presence of calcium chelator BAPTA-AM (EC + BA), PLC inhibitor U73122 (EC+U73122) or its inactive analog U73343 (EC+U73343). Newly invaded rings were scored by Giemsa staining. Invasion rates in RPMI1640 medium in absence of any calcium modulating agents were used as controls to determine invasion inhibition rates in presence of BAPTA-AM, U73122 and U73343.

**Figure 5:**
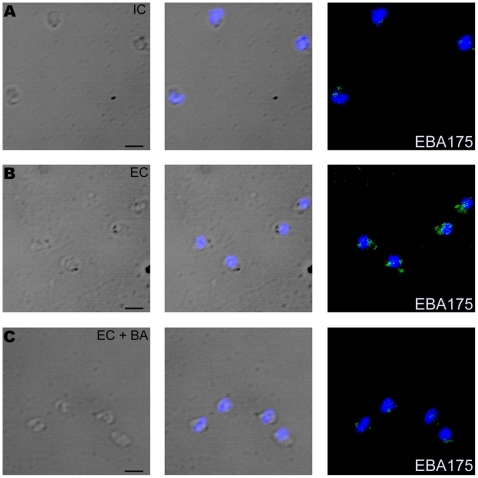
Expression of EBA175 on merozoite surface detected by immunofluorescence assay (IFA) in response to changes in ionic conditions. Expression of microneme protein EBA175 on surface of *P. falciparum* merozoites isolated in IC buffer (IC) (**A**) or following transfer to EC buffer (EC) (**B**) or following transfer to EC buffer after pre-treatment with BAPTA-AM (EC + BA) (**C**) was detected by IFA using anti-EBA175 rabbit sera followed by FITC-conjugated anti-rabbit IgG goat sera (green). Nuclear DNA was counterstained with DAPI (blue). Bright field, bright field merged with DAPI staining and merged fluorescence images (DAPI and FITC) are shown. Black bar indicates 2 µm.

*P. falciparum* merozoites were isolated in IC buffer, labeled with Fluo-4AM, transferred from IC to EC buffer and observed for changes in cytosolic calcium levels by flow cytometry. Cytosolic calcium levels rise when merozoites are transferred from IC to EC buffer (Figure 4A). Next, we tested whether exposure of merozoites to extracellular ionic conditions can trigger the release of microneme proteins to the merozoite surface. Expression of EBA175 on the surface of merozoites increases upon transfer of merozoites from IC to EC buffer (Figure 4B and Table S2). However, treatment of merozoites with BAPTA-AM prior to transfer from IC to EC buffer blocks both the rise in cytosolic calcium and translocation of EBA175 to the merozoite surface (Figures 4A and 4B, Table S2). The translocation of EBA175 to the surface following transfer of merozoites from IC to EC buffer was confirmed by IFA (Figure 5). Microneme protein AMA1 is also secreted to the merozoite surface following transfer of merozoites from IC to EC buffer (Figure S7). EBA175 released to the merozoite surface in response to change in external ionic conditions appears to be primarily localized at the apical end of merozoites (Figure 5).

Following secretion to the merozoite surface, microneme proteins such as EBA175 and AMA1 are proteolytically cleaved and released in the supernatant. The presence of EBA175 (175kD) and AMA1 (44kD and 48kD fragments) in merozoite supernatants following incubation of merozoites in IC or EC buffers was detected by Western blotting. Levels of EBA175 and AMA1 were significantly higher in supernatants of merozoites incubated in EC buffer compared to IC buffer (Figure S8) confirming that extracellular ionic conditions that mimic those found in blood plasma triggers release of microneme proteins to the merozoite surface.

In order to identify the specific ion responsible for rise in cytosolic calcium and release of microneme proteins, we isolated merozoites in IC buffer, transferred them to IC-K_low_ buffer (5 mM NaCl, 5 mM KCl, 135 mM choline-Cl, 1 mM EGTA) and studied the levels of cytosolic calcium and EBA175 expression on the merozoite surface. Transfer of merozoites from IC to IC-K_low_ buffer, which exposes merozoites to a change in potassium ion concentration alone, triggers an increase in cytosolic calcium levels (Figure 4A) and translocation of EBA175 to the merozoite surface (Figure 4C and Table S2). Exposure of merozoites to low [K^+^] as found in blood plasma thus appears to be a key external signal that triggers a rise in cytosolic calcium and induces discharge of microneme proteins.

Transfer of merozoites from a high [K^+^] to low [K^+^] environment triggers a rise in intracellular calcium and microneme release even in the absence of any extracellular calcium (Figures 4A and 4C) implicating internal stores as the potential source of free calcium. Release of calcium from internal stores such as the endoplasmic reticulum (ER) usually involves phospholipase C (PLC) [31], which when activated cleaves phosphatidyl inositol bisphosphate (PIP_2_) to produce diacylglycerol (DAG) and inositol trisphosphate (IP_3_). Binding of IP_3_ to IP_3_ receptor on the ER can stimulate opening of calcium channels and release of free calcium from the ER to the cytoplasm [32]. Although IP_3_ receptor has not been identified in *P. falciparum*, pharmacological evidence suggests that it is likely to be present in Apicomplexan parasites [33]. The *P. falciparum* genome encodes a putative PLC and the PLC inhibitor U73122 has been shown to block *P. falciparum* blood-stage development [8],[34],[35]. We tested whether U73122 can block the release of calcium and translocation of microneme proteins when merozoites are transferred from IC to EC buffer. Merozoites isolated in IC buffer were treated either with U73122 [8],[34],[35] or its inactive analog U73343 [8],[34],[35] prior to transfer to EC buffer. Treatment with U73122 blocks the rise in cytosolic calcium and translocation of EBA175 to the merozoite surface upon transfer from IC to EC buffer (Figures 4A and 4D, Table S2). On the other hand, its inactive analog, U73343 [8],[34],[35], does not inhibit the rise in cytosolic calcium or translocation of EBA175 to the merozoite surface (Figures 4A and 4D, Table S2). Treatment of merozoites with U73122 also blocks erythrocyte invasion by *P. falciparum* merozoites *in vitro* (Figure 4E).

### Interaction of EBA175 with its receptor, glycophorin A, restores basal intracellular calcium levels and triggers release of rhoptry proteins

**Figure 6:**
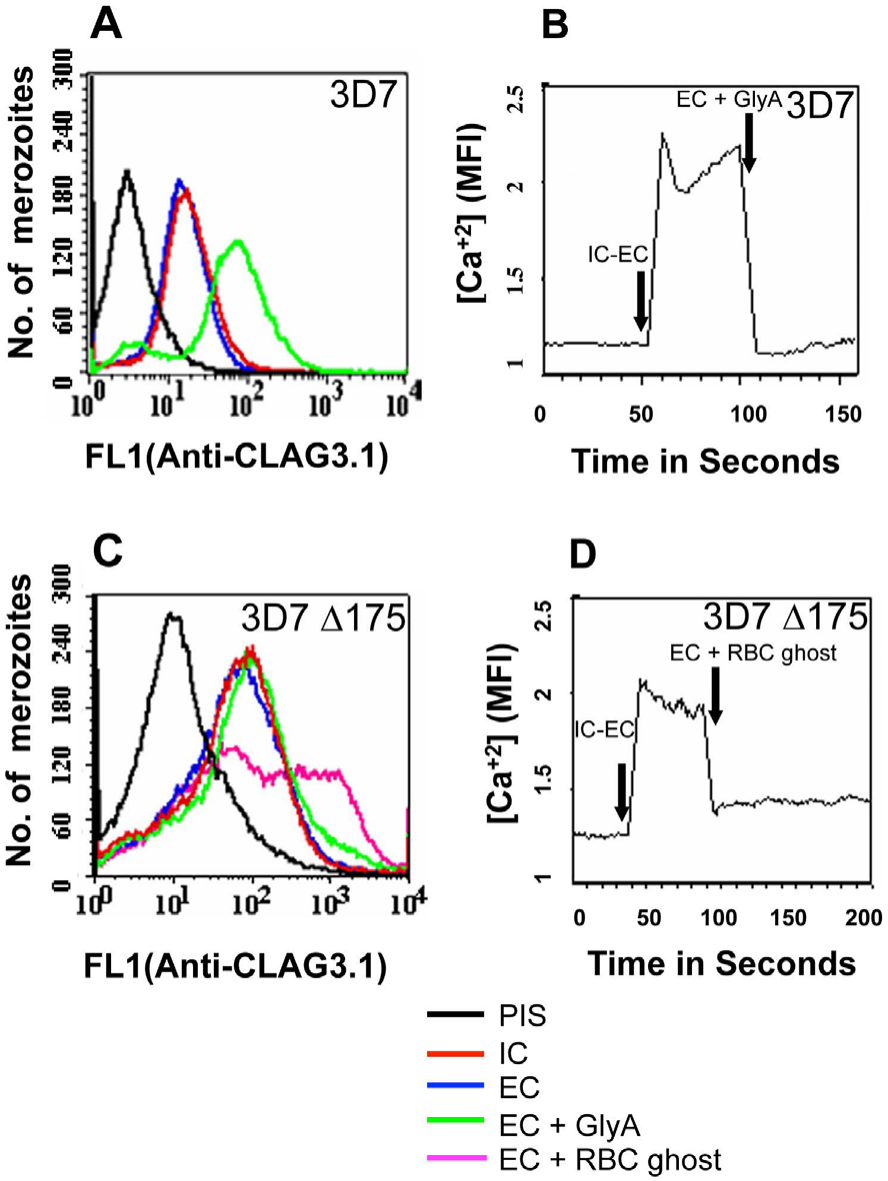
Cytosolic calcium levels and translocation of rhoptry protein CLAG3.1 to merozoite surface in response to interaction with glyA. (**A**) *Expression of CLAG3.1 on merozoite surface following interaction with glyA*. Expression of CLAG3.1 was detected on surface of *P. falciparum* merozoites isolated in buffer mimicking intracellular conditions (IC – 5 mM NaCl, 140 mM KCl, 1 mM EGTA, red), or after transfer from IC buffer to buffer mimicking extracellular conditions (EC - 140 mM NaCl, 5 mM KCl, 1 mM CaCl_2_, blue) or after transfer from IC buffer to EC buffer containing glyA (EC + GlyA, green) using specific sera by flow cytometry. Merozoites isolated in IC buffer and stained with pre-immune sera (PIS, black) were used as controls. (**B**) *Changes in cytosolic calcium levels in* P. falciparum *3D7 merozoites following transfer from intracellular to extracellular ionic conditions and following engagement with glyA*. *P. falciparum* 3D7 merozoites were isolated in IC buffer. Merozoites were labeled with Fluo-4AM and cytosolic calcium levels [Ca^+2^] were measured by flow cytometry before and after transfer from IC to EC buffer (IC-EC) and from EC buffer to EC buffer containing glyA (EC + GlyA). (**C**) *Translocation of CLAG3.1 to surface of* P. falciparum *3D7Δ175 merozoites following receptor engagement*. Expression of CLAG3.1 was detected on surface of *P. falciparum* 3D7Δ175 merozoites isolated in IC buffer (IC, red), after transfer from IC buffer to EC buffer (EC, blue), after transfer from IC buffer to EC buffer containing glyA (EC + GlyA, green) or after transfer from IC buffer to EC buffer containing red blood cell (RBC) ghosts (EC + RBC ghosts, pink) using specific sera by flow cytometry. *P. falciparum* 3D7Δ175 merozoites in IC buffer stained with pre-immune sera (PIS, black) were used as control. (**D**) *Changes in cytosolic calcium levels in* P. falciparum *3D7Δ175 merozoites after transfer from intracellular to extracellular conditions and following binding to erythrocyte ghosts*. *P. falciparum* 3D7Δ175 merozoites were isolated in IC buffer. Merozoites were labeled with Fluo-4AM and cytosolic calcium levels were measured by flow cytometry. Merozoites were transferred first from IC buffer to EC buffer (IC-EC) and from EC buffer to EC buffer containing red blood cell ghosts (EC + RBC ghosts). Mean fluorescence intensities (MFI), which reflect relative levels of free cytosolic calcium measured by flow cytometry are shown as a function of time in seconds (**B**, **D**). Arrows indicate time-points at which merozoites were transferred from IC buffer to EC buffer (IC-EC) (**B**, **D**) or from EC buffer to EC buffer containing gly A (EC + Gly A) (**B**) or from EC buffer to EC buffer containing RBC ghosts (EC + RBC ghosts) (**D**).

**Figure 7:**
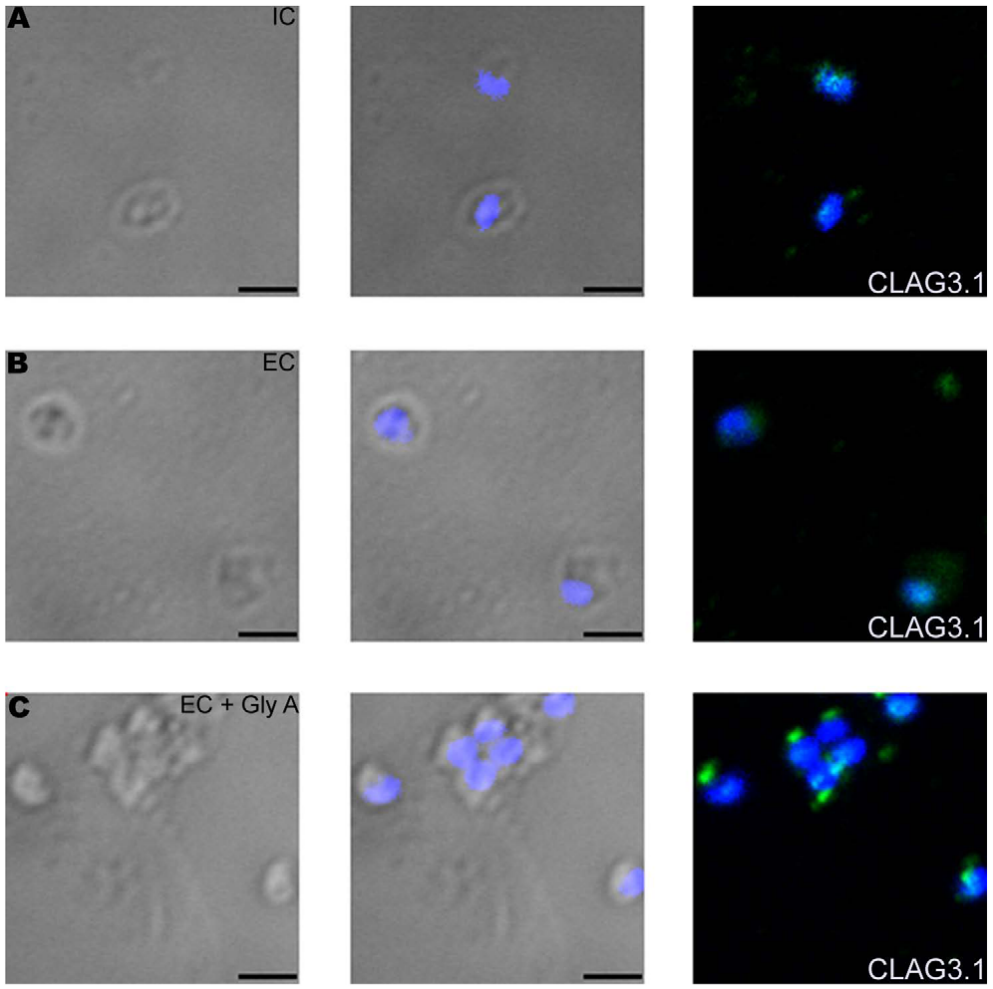
Expression of CLAG3.1 on merozoite surface detected by immunofluorescence assay (IFA) in response to interaction with glyA. Expression of rhoptry protein CLAG3.1 on surface of *P. falciparum* merozoites isolated in IC buffer (IC) (**A**) or following transfer to EC buffer (EC) (**B**) or following transfer to EC buffer containing glyA (EC + GlyA) (**C**) detected by IFA using anti-CLAG3.1 rabbit sera followed by FITC-conjugated (green) anti-rabbit IgG goat sera. Nuclear DNA was counterstained with DAPI (blue). Bright field, bright field merged with DAPI staining and merged fluorescence images (DAPI and FITC) are shown. Black bar indicates 2 µm.

While transfer of merozoites from IC to EC buffer triggers a rise in cytosolic calcium and release of microneme proteins, there is no increase in surface expression of rhoptry proteins such as CLAG3.1 (Figure 6A and Table S3) and PfRH2b (Figure S9) indicating that translocation of microneme and rhoptry proteins is likely to be triggered by distinct external signals. Given that microneme proteins such as EBA175, EBA140 and EBA181, bind erythrocyte receptors during invasion [2], we wondered whether receptor-engagement by these parasite ligands following translocation to the merozoite surface might provide the signal for secretion of rhoptry proteins. In order to test this hypothesis, we transferred *P. falciparum* merozoites from IC buffer to EC buffer containing glyA, the receptor for EBA175, and studied expression of CLAG3.1 on the merozoite surface. There is an increase in surface expression of CLAG3.1 when merozoites are transferred from IC buffer to EC buffer containing glyA (Figure 6A and Table S3). The translocation of CLAG3.1 to the merozoite surface following transfer of merozoites to EC buffer containing glyA was confirmed by IFA using anti-CLAG3.1 sera (Figure 7). CLAG3.1 is localized to the apical end following release to the merzoite surface (Figure 7). Similarly, transfer of merozoites from IC buffer to EC buffer containing glyA also triggers release of rhoptry proteins Rh2b to the merozoite surface (Figure S9). Importantly, cytosolic calcium levels drop when merozoites are transferred to EC buffer containing glyA (Figure 6B). The release of CLAG3.1 to the surface when merozoites are transferred from IC buffer to EC buffer containing glyA was confirmed by IFA (Figure 7). These observations suggest that glyA provides the external signal for restoration of basal intracellular calcium levels and release of rhoptry proteins presumably by interaction with its ligand EBA175. Indeed, when *P. falciparum* 3D7Δ175 merozoites, which have a deletion in the gene encoding EBA175 [36], are transferred from IC buffer to EC buffer containing glyA no increase in surface expression of CLAG3.1 is detected (Figure 6C and Table S3). *P. falciparum* 3D7Δ175 merozoites express EBA175 homologs such as EBA140 and EBA181. EBA140 binds glycophorin C (glyC) whereas EBA181 binds an as yet unidentified trypsin-resistant erythrocyte receptor [2]. EBA140 expression on the merozoite surface increases upon transfer of *P. falciparum* 3D7 and 3D7Δ175 merozoites from IC to EC buffer (Figure S10). Since purified glyC is not available, we tested whether red blood cell (RBC) ghosts, which contain glyC, can trigger rhoptry release in *P. falciparum* 3D7Δ175 merozoites. Transfer of *P. falciparum* 3D7Δ175 merozoites from IC buffer to EC buffer containing RBC ghosts triggers the release of rhoptry protein CLAG3.1 to the merozoite surface (Figure 6C and Table S3). Moreover, cytosolic calcium is restored to basal levels following transfer of *P. falciparum* 3D7Δ175 merozoites to EC buffer containing RBC ghosts (Figure 6D).

## Discussion

Parasites that belong to the phylum Apicomplexa are characterized by the presence of membrane bound organelles at the apical end of their invasive stages. These organelles, called micronemes and rhoptries, contain proteins that play essential roles in the process of host cell invasion. The regulated release of these apical organelles is critical for successful completion of host cell invasion. Here, we have identified the external signals that trigger the co-ordinated release of microneme and rhoptry proteins in *P. falciparum* merozoites during erythrocyte invasion.

We first investigated changes in levels of intracellular calcium in *P. falciparum* merozoites during erythrocyte invasion. Fluo-4AM labeled merozoites that are released in RPMI medium following rupture of mature schizonts in culture were fluorescent indicating that they had elevated levels of cytosolic calcium (Figures 1 and S2). The cytosolic calcium levels dropped following attachment of merozoites to target erythrocytes prior to invasion (Figures 1 and S2). Similar changes in calcium levels have been observed in *T. gondii* tachyzoites during host cell invasion [37].

Given the elevated calcium levels observed in *P. falciparum* merozoites prior to invasion we investigated whether calcium can regulate apical organelle secretion. We developed methods to isolate merozoites, measure levels of cytosolic calcium and study translocation of proteins to the merozoite surface. Treatment of merozoites with calcium ionophore A23187 resulted in elevation of cytosolic calcium (Figure 2A) and increase in expression of microneme proteins such as EBA175 (Figure 2C) on the merozoite surface. The increase in level of EBA175 on the merozoite surface in response to A23187 treatment was also detected by IFA (Figure 3) confirming results obtained by flow cytometry. Pre-treatment of merozoites with the intracellular calcium chelating agent BAPTA-AM blocked rise in cytosolic calcium (Figures 2B) as well as increase in EBA175 on the merozoite surface (Figures 2C and 3). Importantly, elevation of cytosolic calcium levels by A23187 treatment only triggered increased surface expression of microneme proteins. Surface expression of rhoptry proteins such as CLAG3.1 remained unchanged (Figure 2E) suggesting that the signaling pathways responsible for regulated secretion of microneme and rhoptry proteins are likely to be distinct.

Next, we identified a physiologically relevant natural signal that induces a rise in cytosolic calcium to trigger the release of microneme and rhoptry proteins in *P. falciparum* merozoites during invasion. We demonstrated that exposure of merozoites to low [K^+^] as found in blood plasma provided a key environmental signal that released calcium from intracellular stores through a PLC mediated pathway (Figure 3A). The signaling pathways by which exposure to low [K^+^] concentrations leads to a rise in cytosolic calcium remain to be defined. Importantly, transfer of merozoites to a low [K^+^] environment also triggers release of microneme proteins such as EBA175 to the merozoite surface as detected by flow cytometry and IFA (Figures 4 and 5). Moreover, higher levels of EBA175 and AMA1 are detected by Western blotting in supernatants of merozoites incubated in EC buffer compared to IC buffer (Figure S7) confirming that exposure of merozoites to extracellular ionic conditions as found in blood plasma triggers microneme protein release. Confirmation of flow cytometry results by two independent methods, namely, IFA and detection of secreted proteins by Western blotting, validates flow cytometry as a method to quantitatively detect expression of proteins on the merozoite surface.

Following translocation to the surface, binding of EBA175 and its homologues with their erythrocyte receptors triggers the release of rhoptry proteins such as CLAG3.1 (Figures 6 and 7) and PfRH2b (Figure S9). Receptor-engagement by EBA175 and its homologues also restores basal cytosolic calcium levels (Figures 6B and 6D), which may account for the drop observed in merozoites just prior to invasion by video microscopy (Figures 1 and S2). The drop in cytosolic calcium levels following receptor engagement may provide a feedback loop to switch off further secretion of microneme proteins to the merozoite surface.

The ionic environment within parasitized erythrocytes is influenced by the expression of parasite derived new permeation pathways following infection [38]. As a result, the Na^+^/K^+^ composition of the erythrocyte cytosol at late schizont stage is thought to be similar to that found in blood plasma [30],[39]. Moreover, the parasitophorous vacuolar membrane (PVM) is predicted to be freely permeable to ions in the schizont stage [39]. Merozoites developing within schizonts are thus likely to be exposed to a low [K^+^] environment prior to schizont rupture. Our model suggests that microneme proteins such as EBA175 and AMA1 may already be localized on the merozoite surface by the time of schizont rupture and merozoite egress. Indeed, AMA1 has been detected both in micronemes and on the surface of merozoites at the time of release from mature schizonts [40]. Following egress, as merozoites encounter uninfected erythrocytes, the interaction of parasite ligands such as EBA175 with their erythrocyte receptors such as glyA provides the signal for rhoptry release.

It has been previously reported that transgenic parasites that express truncated EBA175 with a deletion of the cytoplasmic domain can not use glyA as a receptor for invasion [41]. Phosphorylation of the cytoplasmic domain of EBA175 may be involved in signal transduction following receptor-engagement. Site-directed mutagenesis of two tyrosines present in the cytoplasmic tail, which are predicted to be phospohorylated, did not, however, result in any change in invasion efficiency [41]. Alternative phosphorylation sites in the cytoplasmic tail of EBA175 need to be investigated. Phosphorylation of the cytoplasmic domain of microneme protein AMA1 is functionally important for successful invasion of host cells [40]. In case of *T. gondii*, a conditional knock-out of AMA-1 results in a defect in rhoptry secretion [42]. In addition, deletion of the cytoplasmic domain of *T. gondii* microneme protein 8 (MIC8) also impairs secretion of rhoptry proteins during invasion [43]. These observations are in line with the induction of rhoptry secretion by EBA175 receptor-engagement reported here. The cytoplasmic domains of microneme proteins are likely to play important functional roles in signal transduction pathways that trigger rhoptry release during invasion.

This study opens the path for analysis of signal transduction pathways that mediate apical organelle release during invasion. Small molecules that target such pathways will block surface translocation of key parasite proteins needed for receptor-binding and may impair erythrocyte invasion. Indeed, we have demonstrated that treatment of *P. falciparum* merozoites with the PLC inhibitor U73122 blocks translocation of microneme proteins and inhibits erythrocyte invasion. Signaling pathway components that are involved in apical organelle release should therefore serve as attractive drug targets.

This study defines the sequence of apical organelle release during invasion. Our model suggests that surface translocation of microneme proteins precedes release of rhoptry proteins during invasion. Indeed, we have demonstrated that receptor engagement by microneme proteins such as EBA175 is required for release of rhoptry proteins. Targeting both microneme and rhoptry proteins with antibodies will thus block distinct steps in the invasion process and may provide a synergistic effect to inhibit erythrocyte invasion efficiently. Identification of key microneme and rhoptry proteins that play crucial roles in invasion should enable the development of efficacious multi-component blood-stage malaria vaccines that target distinct steps in the invasion process to block erythrocyte invasion efficiently and provide protection against malaria.

## Materials and Methods

### *P. falciparum* strain and *in vitro* culture

*P. falciparum* 3D7 was cultured in RPMI 1640 (Invitrogen, USA) supplemented with 27.2 mg/L hypoxanthine (Sigma, USA) and 0.5% Albumax I (Invitrogen, USA) (complete RPMI) using O^+^ RBCs in mixed gas environment (5% O_2_, 5% CO_2_ and 90% N_2_) as previously described [44].

### Imaging calcium fluxes in *P. falciparum* merozoites during erythrocyte invasion by confocal microscopy

*P. falciparum* schizonts were purified from synchronized cultures using magnetic columns (Milteny Biotech, Germany), loaded with 2 µM Fluo-4AM (Molecular Probes, USA) for 30 mins at 37°C under mixed gas environment, washed with complete RPMI 1640 and added to uninfected erythrocytes in complete RPMI to allow re-invasion. The Fluo-4AM loaded *P. falciparum* schizont-uninfected erythrocyte suspension was injected into a Dvorak chamber [4] and observed on a Zeiss LSM510 confocal microscope equipped with a temperature controlled stage. Differential interference contrast (DIC) and fluorescence images (505–550 bandpass filter) were captured using a 63×, 1.4 NA lens. 12-bit time-lapse images of invasion events were captured and mean fluorescence intensity (MFI) within regions of interest containing merozoites in the focal plane were determined after background subtraction using Image Pro software.

### Isolation of *P. falciparum* merozoites

*P. falciparum* 3D7 cultures were synchronized by treatment with sorbitol as described previously [45] in at least two successive cycles to obtain tight synchronization. Progress of synchronized *P. falciparum* schizonts was periodically monitored by light microscopy of Giemsa stained smears prepared from culture samples. When majority of infected erythrocytes reached the mature schizont stage with segmented merozoites, cultures were resuspended in complete RPMI or buffer mimicking intracellular ionic conditions (IC buffer – 5 mM NaCl, 140 mM KCl, 1 mM EGTA). Schizonts were allowed to rupture and release merozoites over a period of 1 hour. Cultures containing unruptured schizonts and released merozoites were centrifuged at 2000 rpm on a Sorvall RT7 centrifuge (500g) for 5 mins to separate released merozoites from unruptured schizonts and uninfected erythrocytes. Supernatants containing free merozoites were centrifuged at 5000 rpm on an Eppendorf 5810R centrifuge (3300 g) for 5 mins to collect merozoites. The merozoites were resuspended in RPMI 1640 medium (incomplete RPMI) or IC buffer for use in experiments. Merozoite preparations had less than 0.5% contaminating schizonts. Number of schizonts that ruptured was scored by Giemsa staining culture samples before and after collection period. Merozoite yields were scored by flow cytometry as described below. On average (5 experiments), 1 merozoite was retrieved per 4 ruptured schizonts.

Isolated merozoites were tested in erythrocyte invasion assays to estimate their viability. Isolated merozoites were mixed with liquid counting fluorescent beads (Becton Dickinson, USA) of known concentration and analyzed by flow cytometry on a FACSCalibur (Becton Dickinson, USA) to score merozoite density (number of merozoites per µl). Density of erythrocyte suspension (number of erythrocytes per µl) was scored using a hemocytometer. Approximately 1×10^6^ merozoites were incubated with ∼1×10^7^ erythrocytes in complete RPMI at 37°C for 18–20 hours under mixed gas environment to allow invasion. Invasion rates were determined by scoring the frequency of ring-infected erythrocytes by Giemsa (Sigma, USA) staining. In parallel, invasion assays were set up by incubating ∼1×10^5^ late stage purified *P. falciparum* schizonts with ∼1×10^7^ erythrocytes to allow schizont rupture and re-invasion. Newly invaded ring-infected erythrocytes were scored by Giemsa staining. Invasion rates from the two methods described were compared to determine whether viability of isolated merozoites is comparable to that of merozoites released from schizonts in culture.

### Treatment of *P. falciparum* merozoites with calcium modulating agents and transfer to ionic conditions mimicking intracellular and extracellular conditions prior to analysis of cytosolic calcium levels or surface expression of merozoite proteins

*P. falciparum* merozoites isolated in RPMI 1640 were treated with 10 µM A23187 (Calbiochem, USA) for 15 mins at 37°C with or without pre-treatment with 50 µM BAPTA-AM for 15 mins at 37°C. Merozoites isolated in IC buffer were transferred to EC buffer, IC-K_low_ buffer (5 mM NaCl, 5 mM KCl, 135 mM choline-Cl, 1 mM EGTA), EC w/o Ca^+2^ buffer (140 mM NaCl, 5 mM KCl, 1 mM EGTA), EC buffer containing glyA (1 mg/ml; Sigma, USA ) or EC buffer containing red blood cell (RBC) ghosts with or without prior treatment with calcium modulators BAPTA-AM (50 µM; Calbiochem, USA), U73122 (10 µM; Calbiochem, USA) or its inactive analog U73343 (10 µM; Calbiochem, USA) for 15 mins at 37°C. RBC ghosts were prepared as follows. RBCs were lysed by resuspending RBC pellets in lysis buffer (cold, 10-fold diluted phosphate buffered saline (PBS)). Lysed RBCs were collected by centrifugation at 15,000 g for 5 mins and resuspended again in lysis buffer. This process was repeated 4–5 times to obtain RBC ghosts devoid of hemoglobin. RBC ghosts were finally resuspended in EC buffer and sonicated for 5 cycles with sonicator on or off for 0.5 seconds each before use. Protein content of RBC ghosts was measured by the bicinchoninic acid assay (BCA). RBC ghosts containing 250 µg of protein were added to 200 µl of merozoite suspension to stimulate rhoptry release.

### Determination of free cytosolic calcium levels in *P. falciparum* merozoites by flow cytometry

*P. falciparum* merozoites isolated in complete RPMI or IC buffer as described above were loaded with 10 µM Fluo-4AM for 20 mins at 37°C, washed, resuspended in same buffers and used for experiments within 5 mins. Fluo-4AM loaded *P. falciparum* merozoites were treated with calcium modulating agents and/or transferred from ionic environments mimicking intracellular conditions to extracellular conditions as described above prior to analysis of fluorescence signal on FACSCalibur (Becton Dickinson, USA) using CellQuest software. The Fluo-4AM loaded merozoites were excited at 488 nm and fluorescence signal was detected with a 430/30 nm band pass filter for periods of 2–3 mins. Merozoites were gated on the basis of their forward scatter and side scatter. Histograms of mean fluorescence intensity (MFI), which reflects cytosolic calcium levels in merozoites, were plotted against time using FlowJo software.

### Determination of *in situ* dissociation constants for Fluo-4AM in *P. falciparum* merozoites under intracellular and extracellular ionic conditions

*In situ* dissociation constants for Fluo-4AM were measured in *P. falciparum* merozoites under different ionic conditions using methods described earlier for mammalian cells [46]. Calcium Calibration Buffer Concentrate Kits (Molecular Probes, USA) containing 100 mM Ca-EGTA, pH 7.2 and 100 mM K_2_EGTA, pH 7.2 were used to generate IC and EC buffers containing a range of known concentrations of free calcium (17 nM to 39 µM) as described by the manufacturer. Merozoites were loaded with 10 µM Fluo-4AM for 20 mins at 37°C and washed with IC or EC buffers. Loaded merozoites were resuspended in IC and EC buffers containing range of calcium concentrations. Ionomycin (10 µM) (Calbiochem, USA) was added to the merozoite suspension to equilibrate intracellular and extracellular calcium concentrations. The fluorescence signal was measured at 485 nM using a Perkin Elmer Victor^3^ fluorimeter. Measured fluorescence intensity was used to plot Log[(F – F_min_)/F_max_ – F)] against Log[Ca^+2^_free_] where F is the fluorescence intensity at different known free calcium concentrations, F_min_ is the fluorescence intensity at zero free calcium concentration and F_max_ is the fluorescence intensity at saturating free calcium concentration (39 µM free Ca^+2^). The x-intercept of the plot is Log K_d_ of Fluo-4AM. The K_d_ values determined represent affinity of Fluo-4 for calcium in the merozoite cytoplasm under ionic conditions that mimic intracellular and extracellular ionic environments.

### Detection of proteins on *P. falciparum* merozoite surface by flow cytometry and fluorescence microscopy

*P. falciparum* merozoites isolated in complete RPMI or IC buffer were treated with calcium modulating agents and/or transferred from ionic environments mimicking intracellular conditions to extracellular conditions as described above prior to analysis of expression of microneme proteins (EBA175 and AMA1), rhoptry proteins (CLAG3.1 and RH2b) or merozoite surface protein (MSP4) on the merozoite surface. Merozoites were fixed with 0.15% chilled p-formaldehyde for 1 hour and used for detection of parasite proteins on the merozoite surface. Fixed merozoites were washed with PBS, incubated with sera raised against EBA175 (mouse sera at 1∶50), AMA1 (rabbit sera at 1∶50 dilution), CLAG3.1 (rabbit sera at 1∶50 dilution), PfRH2b (rabbit sera at 1∶50 dilution) and MSP4 (rabbit sera at 1∶50 dilution) for I hour on ice. Merozoites were washed two times with PBS, incubated with fluorescein isothiocyanate (FITC) conjugated anti-mouse IgG (Sigma, USA) or FITC conjugated anti-rabbit IgG (Sigma, USA) for I hour on ice, washed two times with PBS and used for analysis by flow cytometry using a Becton Dickinson FACS Callibur. Merozoites were excited at 488 nm and fluorescence signal was detected with a 430/30 nm band pass filter. Merozoites were gated on the basis of their forward scatter and side scatter. The gain of FL1 channel was set to ensure that fluorescence intensities of merozoite samples stained with pre-immune sera as well as immune sera against candidate proteins fall within the window of 10^0^ to 10^4^. In each experiment, data was acquired for 50,000 merozoites with the same FACS settings for each condition. FACS settings for independent experiments were not always identical. Data was analyzed using CellQuest software. Histograms showing logarithmic green fluorescence intensities (FL1) were plotted for each sample. Normalized relative MFI values from three independent experiments are reported for each condition to demonstrate reproducibility of the results. Merozoites were also labeled with mouse sera raised against the cytoplasmic protein NAPL at 1∶50 dilution as control. Merozoites were permeabilized by adding 0.05% saponin to primary and secondary sera to detect intracellular proteins. Expression of EBA175, CLAG3.1 and NAPL was also analyzed by IFA using a Nikon TE 300 fluorescence microscope following staining with primary and secondary sera as described above. Nuclear DNA was counterstained with 40 nM 4′,6-diamidino-2-phenylindole (DAPI; Molecular Probes, USA) in IFA.

### Detection of proteins secreted from *P. falciparum* merozoites by Western blotting

*P. falciparum* merozoites isolated in IC buffer were incubated in either IC or EC buffer for 15 minutes at 37°C with or without prior treatment with BAPTA-AM for 20 mins at 37°C to evaluate the role of ionic conditions in secretion of microneme proteins. Merozoite supernatants were separated by centrifugation and used for detection of microneme proteins, EBA175 and AMA1, by Western blotting using specific rabbit antisera. Anti-EBA175 rabbit sera were used at a dilution of 1∶200, anti-AMA1 rabbit sera were used at 1∶2,000 dilution and horse radish peroxidase (HRP) conjugated anti-rabbit IgG goat sera (Sigma, USA) were used at a dilution of 1∶2,000 for Western blotting. Anti-NAPL rabbit sera were used at a dilution of 1∶2,000 as control for Western blotting with merozoite pellets as well as with merozoite supernatants for each condition. Densitometry analyses of Western blots of merozoite pellets and supernatants using anti-NAPL sera were used to normalize levels of EBA175 and AMA1 detected in merozoite supernatants under different conditions.

### Measuring effect of calcium modulating agents on erythrocyte invasion by *P. falciparum* merozoites

Merozoites isolated from 2 ml of *P. falcipaum* 3D7 culture with 5% parasitemia were mock-treated with RPMI 1640 or treated with 1 mM BAPTA-AM (Calbiochem, USA), 10 µM U73122 (Calbiochem, USA) or 10 µM U73343 (Calbiochem, USA) for 15 mins at 37°C, washed with IC buffer, resuspended in EC buffer and incubated with 1×10^7^ erythrocytes in EC buffer at 37°C under mixed gas environment for 2 hours to allow invasion. EC buffer was then replaced with complete RPMI. After 18–20 hours of incubation in complete RPMI under mixed gas environment to allow development of ring stages, the percentage of infected erythrocytes was scored by microscopy of Giemsa stained smears to determine invasion rates.

## Supporting Information

Figure S1 Distribution of calcium-sensitive fluorescence indicator Fluo-4AM in *P. falciparum* merozoites. A–B. *P. falciparum* merozoites were isolated in complete RPMI, loaded with Fluo-4AM and observed by confocal microscopy. Bright field (A) and fluorescence images (B) are shown. Fluorescence signal indicates that Fluo-4AM is uniformly distributed in the merozoite cytoplasm. Scale bar indicates 10 µm. C–F. *P. falciparum* merozoites were isolated in complete RPMI, loaded with Fluo-4AM, counterstained with DAPI and observed by confocal microscopy. Bright field (C) and fluorescence images (D–F) are shown. Images showing DAPI staining (D), Fluo-4AM fluorescence (E) and merge (F) indicate that Fluo-4AM is uniformly distributed in the merozoite cytoplasm. Scale bar indicates 2 µm.
(6.68 MB PDF)

Figure S2 Cytosolic calcium levels in *P. falciparum* merozoites during invasion of erythrocytes. *P. falciparum* late stage schizonts were purified, labeled with Fluo-4AM and added to erythrocytes to allow re-invasion. Calcium levels were monitored in merozoites during invasion by time-lapse video microscopy. Both DIC and fluorescence images were acquired at 2 second intervals. Mean fluorescence intensities (MFI) after background subtraction are reported for three merozoites that complete invasion and three merozoites that do not invade erythrocytes in the same time period (A–C).
(6.85 MB PDF)

Figure S3 Detection of NAPL in *P. falciparum* merozoites by flow cytometry. *P. falciparum* merozoites were isolated in complete RPMI and fixed with p-formaldehyde. Specific sera were used to detect cytoplasmic protein NAPL in either p-formaldehyde fixed merozoites (A) or pformaldehyde fixed merozoites permeabilized with 0.05% saponin (B) using anti-NAPL rabbit sera (Red) by flow cytometry. Merozoites stained with preimmune serum (PIS) were used as control (Black). NAPL was only detected in permeabilized merozoites.
(3.55 MB PDF)

Figure S4 Detection of NAPL in *P. falciparum* merozoites by IFA. *P. falciparum* merozoites were isolated in complete RPMI and fixed with pformaldehyde. Specific sera were used to detect cytoplasmic protein NAPL in either p-formaldehyde fixed merozoites (A–D) or p-formaldehyde fixed merozoites permeabilized with 0.05% saponin (E–F) by IFA using anti-NAPL rabbit sera. Merozoites were counterstained with DAPI. (A) shows bright field image, (B) shows DAPI staining, (C) shows staining with anti-NAPL sera and (D) shows merge of (B) and (C). (E) shows bright field image, (F) shows DAPI staining, (G) shows staining with anti-NAPL sera and (H) shows merge of (F) and (G). Scale bar indicated 2 µm.
(4.67 MB PDF)

Figure S5 Surface expression of AMA1 following treatment with calcium ionophore A23187. Expression of microneme protein AMA-1 was detected using specific sera on surface of *P. falciparum* merozoites isolated in complete RPMI (RPMI, red) or following treatment with either A23187 (A23187, blue) or BAPTA-AM followed by A23187 (BA + A23187, green) (A). AMA-1 was also detected in merozoites permeabilized with 0.05% saponin using specific sera (B). Untreated merozoites stained with pre-immune serum (PIS, black) were used as controls.
(4.38 MB PDF)

Figure S6 Determination of *in situ* dissociation constants for calcium sensitive fluorescence indicator Fluo-4AM under ionic conditions mimicking intracellular (IC) and extracellular (EC) environments. Fluo-4AM loaded merozoites were resuspended in buffers mimicking IC and EC ionic conditions with a range of free calcium concentrations. Ionomycin (10 µM) was added to equilibrate IC and EC calcium concentrations of merozoites. Measured fluorescence intensity was used to plot Log[(F−F_min_)/(F_max_−F)] against Log([Ca+2]free) where F is the fluorescence intensity at different known free calcium concentrations, F_min_ is the fluorescence intensity at zero free calcium concentration and F_max_ is the fluorescence intensity at saturating free calcium concentration (39 µM). The x-intercepts of the plots provide *in situ* Log K_d_ of Fluo-4AM in IC (A) and EC (B) buffers.
(3.47 MB PDF)

Figure S7 Translocation of microneme protein AMA1 to merozoite surface following exposure to extracellular ionic environment. Expression of AMA1 was detected using specific sera by flow cytometry on surface of merozoites isolated in buffer mimicking intracellular conditions (IC - 5 mM NaCl, 140 mM KCl, 1 mM EGTA, red), after transfer from IC buffer to buffer mimicking extracellular conditions (EC - 140 mM NaCl, 5 mM KCl, 1 mM CaCl_2_, blue) or following transfer from IC to EC buffer after prior treatment with BAPTA-AM (EC + BA, green). Merozoites in IC buffer stained with preimmune sera were used as controls (PIS, black).
(1.42 MB PDF)

Figure S8 Detection of EBA175 and AMA1 in supernatants following exposure of merozoites to extracellular ionic environment by Western blotting. Secretion of microneme proteins EBA175 (A) and AMA1 (B) was detected by Western blotting using specific sera in supernatants of *P. falciparum* merozoites (EBA175 (sup), AMA1 (sup)) incubated in buffer mimicking intracellular conditions (IC - 5 mM NaCl, 140 mM KCl, 1 mM EGTA), buffer mimicking extracellular conditions (EC - 140 mM NaCl, 5 mM KCl, 1 mM CaCl_2_) or following incubation in EC buffer after prior treatment with BAPTA-AM (EC + BA). NAPL was detected in merozoite pellets (NAPL (pellet)) and merozoite supernatants (NAPL (sup)) using specific sera as loading and lysis controls respectively. Numbers indicate relative intensities measured by densitometry after normalization based on NAPL (pellet) and NAPL (sup) data.
(4.90 MB PDF)

Figure S9 Translocation of rhoptry proteins PfRH2b to merozoite surface in response to binding with glyA. Expression of PfRH2b was detected using specific sera by flow cytometry on surface of merozoites isolated in buffer mimicking intracellular conditions (IC - 5 mM NaCl, 140 mM KCl, 1 mM EGTA, red), after transfer from IC buffer to buffer mimicking extracellular conditions (EC - 140 mM NaCl, 5 mM KCl, 1 mM CaCl_2_, blue) or after transfer from IC buffer to EC buffer containing glyA (EC + glyA, green). Merozoites in IC buffer stained with pre-immune sera were used as controls (PIS, black).
(2.27 MB PDF)

Figure S10 Translocation of EBA140 to the surface of *P. falciparum* 3D7 and 3D7Δ175 merozoites in response to changes in ionic conditions. Expression of EBA-140 was detected using specific sera by flow cytometry on surface of *P. falciparum* 3D7 (A) and 3D7Δ175 merozoites (B) isolated in IC buffer (IC, red), or following transfer from IC buffer to EC buffer (EC, blue). Merozoites in IC buffer stained with pre-immune serum were used as controls (PIS, black).
(4.17 MB PDF)

Table S1 Translocation of microneme and rhoptry proteins to the surface of *P. falciparum* 3D7 merozoites following stimulation with calcium ionophore A23187.
(0.04 MB DOC)

Table S2 Translocation of EBA-175 to the surface of *P. falciparum* 3D7 merozoites in response to different ionic conditions.
(0.04 MB DOC)

Table S3 Translocation of rhoptry protein CLAG3.1 to surface of *P. falciparum* 3D7 and 3D7Δ175 merozoites in response to binding with glyA and RBC ghosts.
(0.04 MB DOC)

Video S1 Schizont rupture and erythrocyte invasion by *P. falciparum* merozoites. *P. falciparum* late stage schizonts were labeled with Fluo-4AM and added to erythrocytes to allow re-invasion. Both DIC and fluorescence images were acquired simultaneously on a Zeiss LSM510 confocal microscope at 2 second intervals with a 63×, 1.4 NA lens, using 488 nm excitation at low power to minimize bleaching and cell damage. DIC images are shown.
(3.42 MB MOV)

Video S2 Calcium levels during schizont rupture and erythrocyte invasion by *P. falciparum* merozoites. P. falciparum late stage schizonts were labeled with Fluo-4AM and added to erythrocytes to allow reinvasion. Both DIC and fluorescence images were acquired simultaneously on a Zeiss LSM510 confocal microscope at 2 second intervals with a 63×, 1.4 NA lens, using 488 nm excitation at low power to minimize bleaching and cell damage. Fluorescence images are shown.
(3.42 MB MOV)

Video S3 Calcium levels during schizont rupture and erythrocyte invasion by *P. falciparum* merozoites. *P. falciparum* late stage schizonts were labeled with Fluo-4AM and added to erythrocytes to allow reinvasion. Both DIC and fluorescence images were acquired simultaneously on a Zeiss LSM510 confocal microscope at 2 second intervals with a 63×, 1.4 NA lens, using 488 nm excitation at low power to minimize bleaching and cell damage. A merge of the DIC and fluorescence images is shown.
(3.42 MB MOV)
