# Supplementary figures and images for: Distinct External Signals Trigger Sequential Release of Apical Organelles during Erythrocyte Invasion by Malaria Parasites
Source: PLoS Pathog. 2010 Feb 5;6(2):e1000746. doi: 10.1371/journal.ppat.1000746 (PMC2816683; doi:10.1371/journal.ppat.1000746)

**A**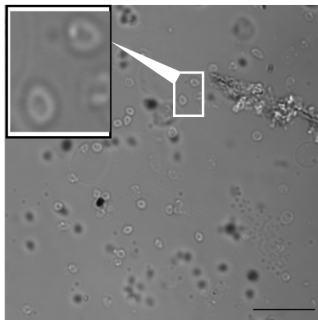**B**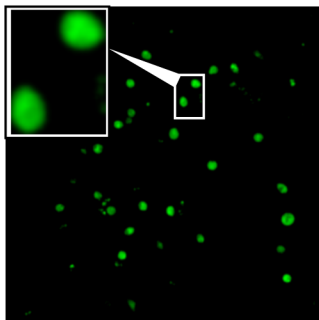**C**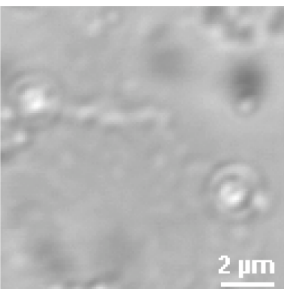**D**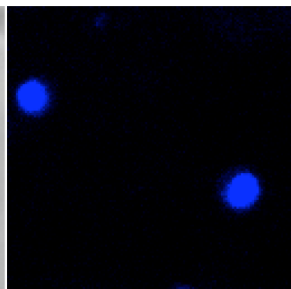**E**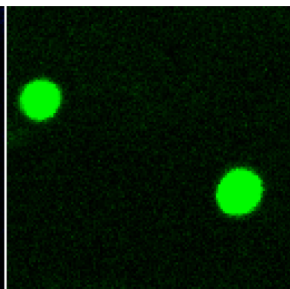**F**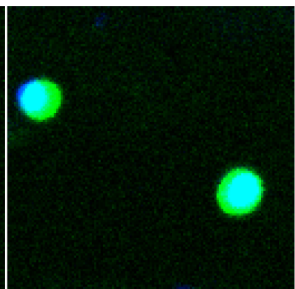

Supplement: Figure S1 — Distribution of calcium-sensitive fluorescence indicator Fluo-4AM in P. falciparum merozoites. A–B. P. falciparum merozoites were isolated in complete RPMI, loaded with Fluo-4AM and observed by confocal microscopy. Bright field (A) and fluorescence images (B) are shown. Fluorescence signal indicates that Fluo-4AM is uniformly distributed in the merozoite cytoplasm. Scale bar indicates 10 µm. C–F. P. falciparum merozoites were isolated in complete RPMI, loaded with Fluo-4AM, counterstained with DAPI and observed by confocal microscopy. Bright field (C) and fluorescence images (D–F) are shown. Images showing DAPI staining (D), Fluo-4AM fluorescence (E) and merge (F) indicate that Fluo-4AM is uniformly distributed in the merozoite cytoplasm. Scale bar indicates 2 µm. (6.68 MB PDF) [file ppat.1000746.s001.pdf]

**A**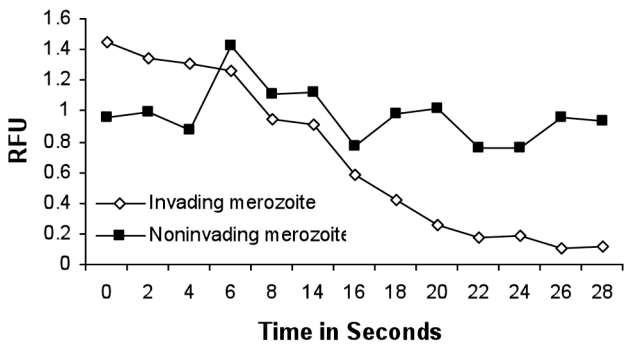**B**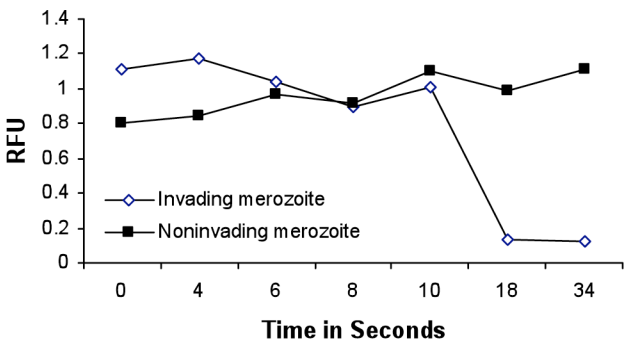**C**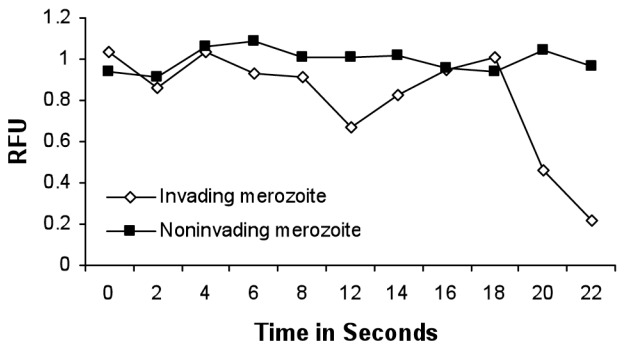

Supplement: Figure S2 — Cytosolic calcium levels in P. falciparum merozoites during invasion of erythrocytes. P. falciparum late stage schizonts were purified, labeled with Fluo-4AM and added to erythrocytes to allow re-invasion. Calcium levels were monitored in merozoites during invasion by time-lapse video microscopy. Both DIC and fluorescence images were acquired at 2 second intervals. Mean fluorescence intensities (MFI) after background subtraction are reported for three merozoites that complete invasion and three merozoites that do not invade erythrocytes in the same time period (A–C). (6.85 MB PDF) [file ppat.1000746.s002.pdf]

**A**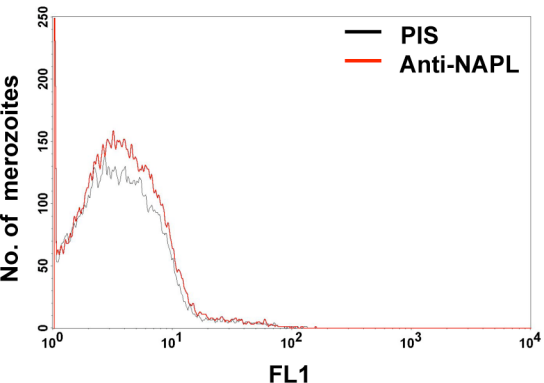**B**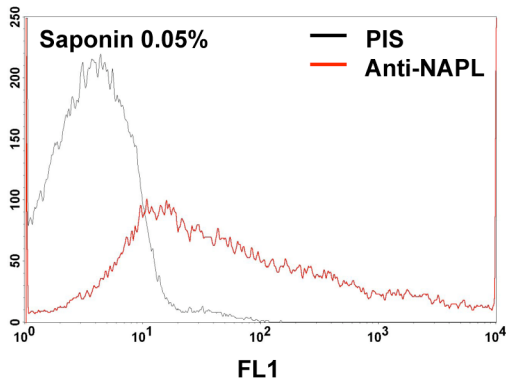

Supplement: Figure S3 — Detection of NAPL in P. falciparum merozoites by flow cytometry. P. falciparum merozoites were isolated in complete RPMI and fixed with p-formaldehyde. Specific sera were used to detect cytoplasmic protein NAPL in either p-formaldehyde fixed merozoites (A) or pformaldehyde fixed merozoites permeabilized with 0.05% saponin (B) using anti-NAPL rabbit sera (Red) by flow cytometry. Merozoites stained with preimmune serum (PIS) were used as control (Black). NAPL was only detected in permeabilized merozoites. (3.55 MB PDF) [file ppat.1000746.s003.pdf]

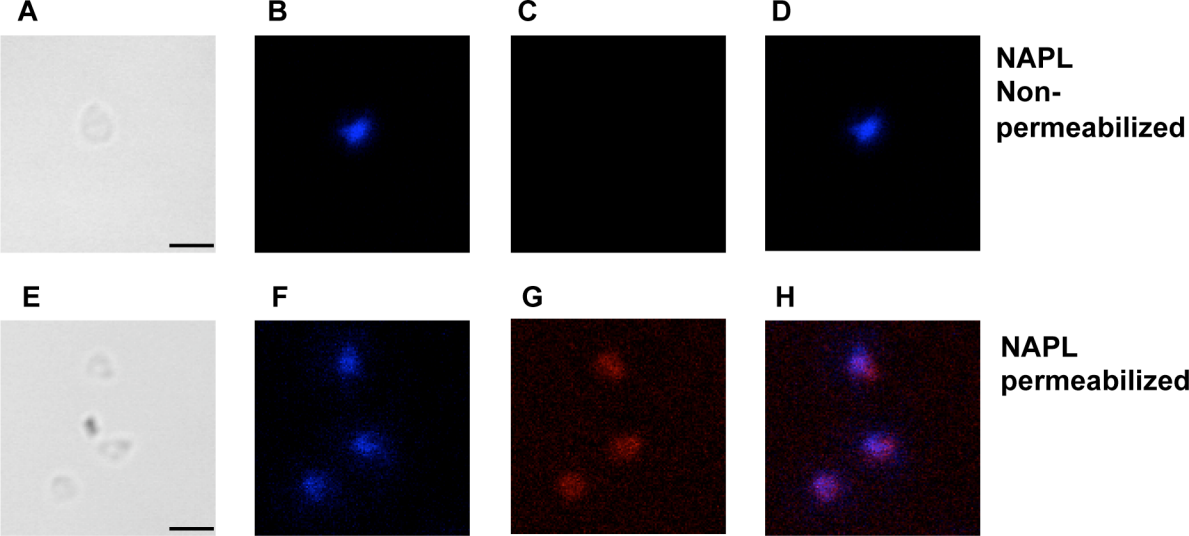

Supplement: Figure S4 — Detection of NAPL in P. falciparum merozoites by IFA. P. falciparum merozoites were isolated in complete RPMI and fixed with pformaldehyde. Specific sera were used to detect cytoplasmic protein NAPL in either p-formaldehyde fixed merozoites (A–D) or p-formaldehyde fixed merozoites permeabilized with 0.05% saponin (E–F) by IFA using anti-NAPL rabbit sera. Merozoites were counterstained with DAPI. (A) shows bright field image, (B) shows DAPI staining, (C) shows staining with anti-NAPL sera and (D) shows merge of (B) and (C). (E) shows bright field image, (F) shows DAPI staining, (G) shows staining with anti-NAPL sera and (H) shows merge of (F) and (G). Scale bar indicated 2 µm. (4.67 MB PDF) [file ppat.1000746.s004.pdf]

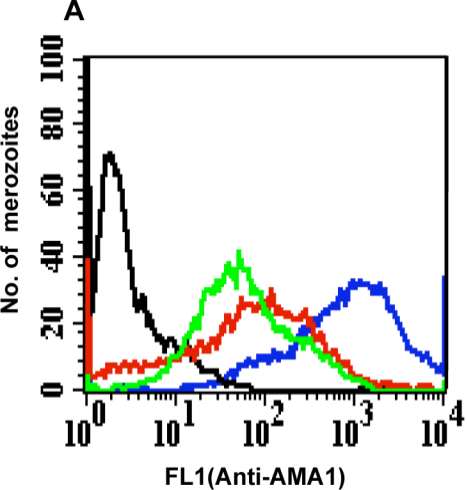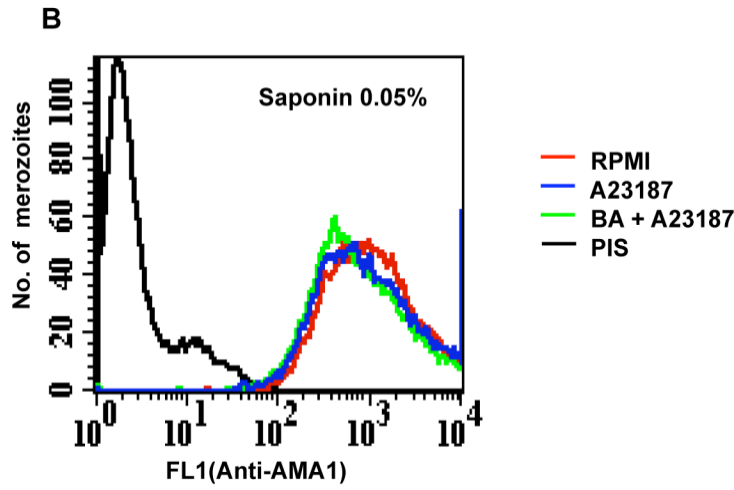

Supplement: Figure S5 — Surface expression of AMA1 following treatment with calcium ionophore A23187. Expression of microneme protein AMA-1 was detected using specific sera on surface of P. falciparum merozoites isolated in complete RPMI (RPMI, red) or following treatment with either A23187 (A23187, blue) or BAPTA-AM followed by A23187 (BA + A23187, green) (A). AMA-1 was also detected in merozoites permeabilized with 0.05% saponin using specific sera (B). Untreated merozoites stained with pre-immune serum (PIS, black) were used as controls. (4.38 MB PDF) [file ppat.1000746.s005.pdf]

**A**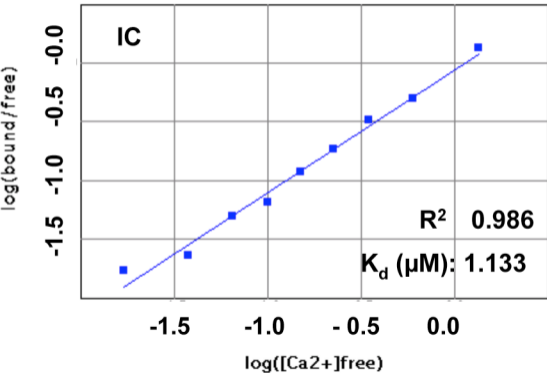**B**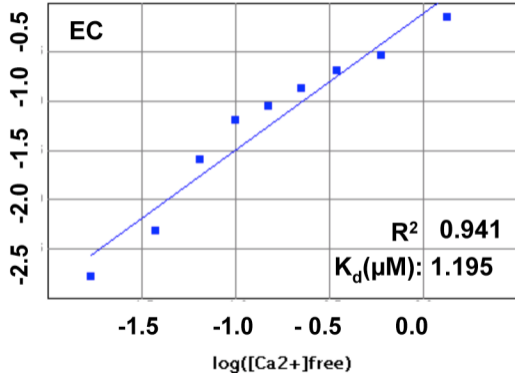

Supplement: Figure S6 — Determination of in situ dissociation constants for calcium sensitive fluorescence indicator Fluo-4AM under ionic conditions mimicking intracellular (IC) and extracellular (EC) environments. Fluo-4AM loaded merozoites were resuspended in buffers mimicking IC and EC ionic conditions with a range of free calcium concentrations. Ionomycin (10 µM) was added to equilibrate IC and EC calcium concentrations of merozoites. Measured fluorescence intensity was used to plot Log[(F−Fmin)/(Fmax−F)] against Log([Ca+2]free) where F is the fluorescence intensity at different known free calcium concentrations, Fmin is the fluorescence intensity at zero free calcium concentration and Fmax is the fluorescence intensity at saturating free calcium concentration (39 µM). The x-intercepts of the plots provide in situ Log Kd of Fluo-4AM in IC (A) and EC (B) buffers. (3.47 MB PDF) [file ppat.1000746.s006.pdf]

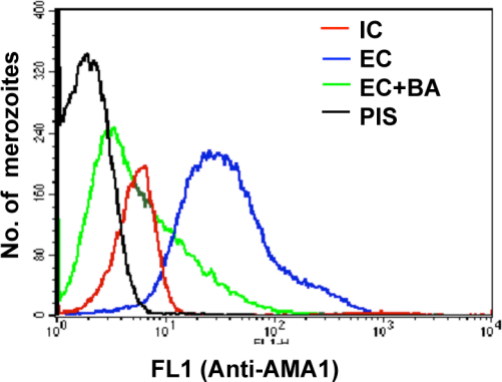

Supplement: Figure S7 — Translocation of microneme protein AMA1 to merozoite surface following exposure to extracellular ionic environment. Expression of AMA1 was detected using specific sera by flow cytometry on surface of merozoites isolated in buffer mimicking intracellular conditions (IC - 5 mM NaCl, 140 mM KCl, 1 mM EGTA, red), after transfer from IC buffer to buffer mimicking extracellular conditions (EC - 140 mM NaCl, 5 mM KCl, 1 mM CaCl2, blue) or following transfer from IC to EC buffer after prior treatment with BAPTA-AM (EC + BA, green). Merozoites in IC buffer stained with preimmune sera were used as controls (PIS, black). (1.42 MB PDF) [file ppat.1000746.s007.pdf]

**A**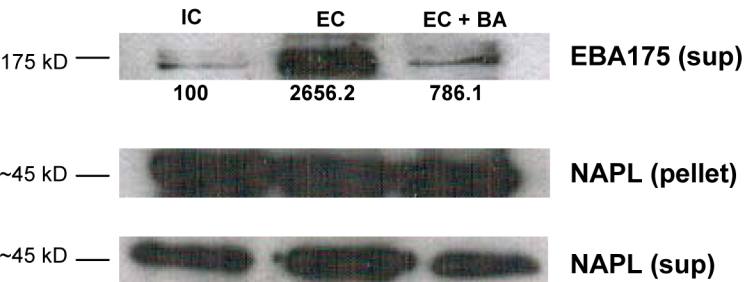**B**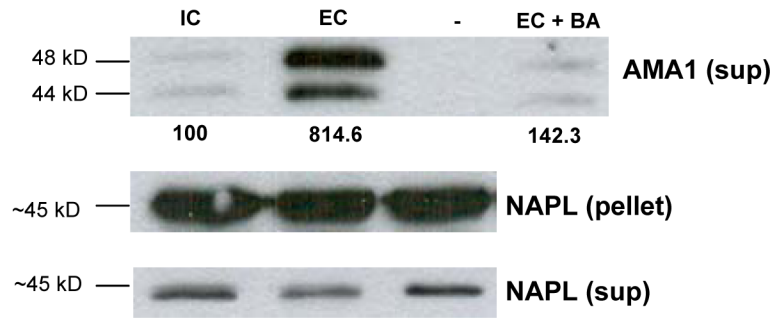

Supplement: Figure S8 — Detection of EBA175 and AMA1 in supernatants following exposure of merozoites to extracellular ionic environment by Western blotting. Secretion of microneme proteins EBA175 (A) and AMA1 (B) was detected by Western blotting using specific sera in supernatants of P. falciparum merozoites (EBA175 (sup), AMA1 (sup)) incubated in buffer mimicking intracellular conditions (IC - 5 mM NaCl, 140 mM KCl, 1 mM EGTA), buffer mimicking extracellular conditions (EC - 140 mM NaCl, 5 mM KCl, 1 mM CaCl2) or following incubation in EC buffer after prior treatment with BAPTA-AM (EC + BA). NAPL was detected in merozoite pellets (NAPL (pellet)) and merozoite supernatants (NAPL (sup)) using specific sera as loading and lysis controls respectively. Numbers indicate relative intensities measured by densitometry after normalization based on NAPL (pellet) and NAPL (sup) data. (4.90 MB PDF) [file ppat.1000746.s008.pdf]

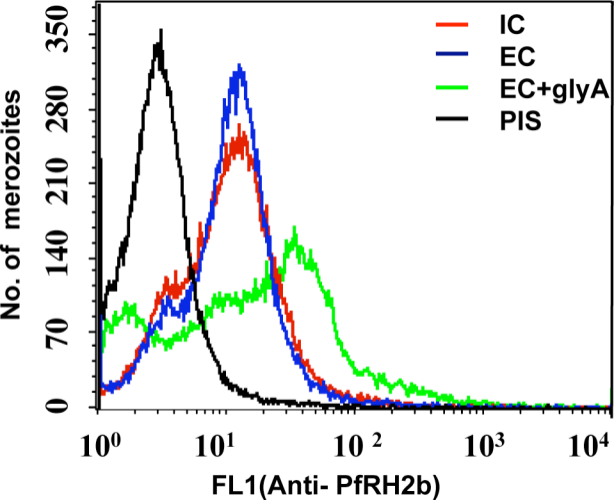

Supplement: Figure S9 — Translocation of rhoptry proteins PfRH2b to merozoite surface in response to binding with glyA. Expression of PfRH2b was detected using specific sera by flow cytometry on surface of merozoites isolated in buffer mimicking intracellular conditions (IC - 5 mM NaCl, 140 mM KCl, 1 mM EGTA, red), after transfer from IC buffer to buffer mimicking extracellular conditions (EC - 140 mM NaCl, 5 mM KCl, 1 mM CaCl2, blue) or after transfer from IC buffer to EC buffer containing glyA (EC + glyA, green). Merozoites in IC buffer stained with pre-immune sera were used as controls (PIS, black). (2.27 MB PDF) [file ppat.1000746.s009.pdf]

A

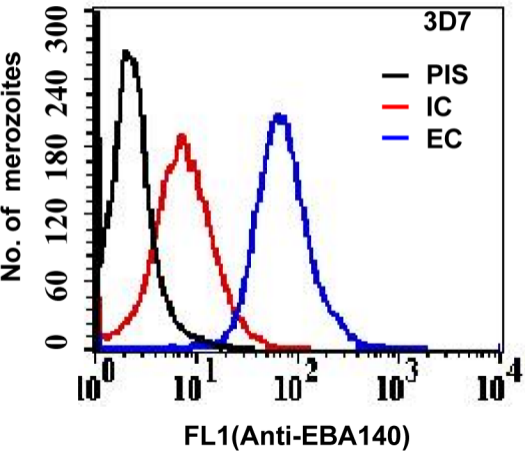

B

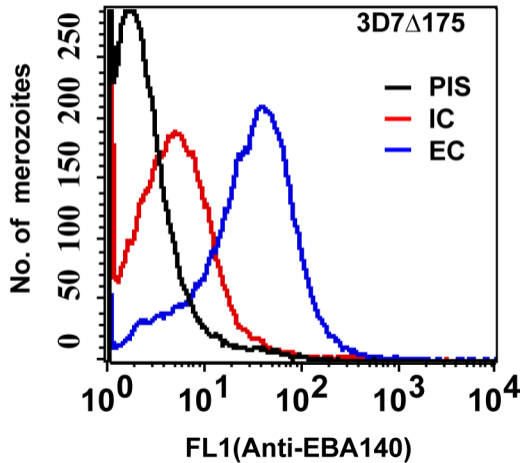

Supplement: Figure S10 — Translocation of EBA140 to the surface of P. falciparum 3D7 and 3D7Δ175 merozoites in response to changes in ionic conditions. Expression of EBA-140 was detected using specific sera by flow cytometry on surface of P. falciparum 3D7 (A) and 3D7Δ175 merozoites (B) isolated in IC buffer (IC, red), or following transfer from IC buffer to EC buffer (EC, blue). Merozoites in IC buffer stained with pre-immune serum were used as controls (PIS, black). (4.17 MB PDF) [file ppat.1000746.s010.pdf]
