# Supplementary material for: Distinct External Signals Trigger Sequential Release of Apical Organelles during Erythrocyte Invasion by Malaria Parasites
Source: PLoS Pathog. 2010 Feb 5;6(2):e1000746. doi: 10.1371/journal.ppat.1000746 (PMC2816683; doi:10.1371/journal.ppat.1000746)
Supplement: Table S1 — Translocation of microneme and rhoptry proteins to the surface of P. falciparum 3D7 merozoites following stimulation with calcium ionophore A23187. (0.04 MB DOC) [file ppat.1000746.s011.doc]

**Supplementary Table S1.** **Translocation of microneme and rhoptry proteins to the surface of *P. falciparum 3D7* merozoites following stimulation with calcium ionophore A23187.**

| Treatment | Relative MFI for Surface Expression of Parasite Proteins^+^(Avg + SD) N = 3 | | | | |
| --- | --- | --- | --- | --- | --- |
|  | **EBA175** | **EBA175*** | **AMA1** | **CLAG3.1** | **MSP4** |
| RPMI | 100 | 100 | 100 | 100 | 100 |
| RPMI+A23187 | 502.8 ± 15.4 | 104.4 ± 3.9 | 1297.3 ± 97.0 | 114.9 ± 6.3 | 102.7 ± 5.2 |
| RPMI+BA+A23187 | 91.9 ± 6.4 | 99.9 ± 1.1 | 140.8 ± 15.7 | 109.7 ± 3.7 | 104.9 ± 0.6 |

MFI: Mean Fluorescence Intensity

^+^MFI values for staining of merozoites with antibodies against EBA175, AMA1, CLAG3.1 and MSP4 in RPMI were normalized to 100. MFI values for staining of merozoites with antibodies against EBA175, AMA1, CLAG3.1 and MSP4 under other conditions are reported relative to MFI for staining of merozoites in RPMI.

EBA175*: Intracellular staining with anti-EBA175 rabbit sera in presence of 0.05% saponin

BA: BAPTA-AM

N = 3 independent experiments
