# Supplementary material for: Distinct External Signals Trigger Sequential Release of Apical Organelles during Erythrocyte Invasion by Malaria Parasites
Source: PLoS Pathog. 2010 Feb 5;6(2):e1000746. doi: 10.1371/journal.ppat.1000746 (PMC2816683; doi:10.1371/journal.ppat.1000746)
Supplement: Table S2 — Translocation of EBA-175 to the surface of P. falciparum 3D7 merozoites in response to different ionic conditions. (0.04 MB DOC) [file ppat.1000746.s012.doc]

| Treatment | Relative MFI for Surface Expression of EBA175^*^(Avg + SD)N=3 |
| --- | --- |
| **IC** | 100 |
| **EC** | 387.0 ± 47.0 |
| **IC-K _low_** | 349.3 ± 35.9 |
| **EC+BA** | 75.9 ± 13.4 |
| **EC+U73122** | 77.4 ± 25.2 |
| **EC+U73343** | 364.2 ± 43.7 |

**Supplementary Table S2. Translocation of EBA-175 to the surface of *P. falciparum* 3D7 merozoites in response to different ionic conditions.**

MFI: Mean Fluorescence Intensity

^*^MFI values for staining of merozoites with anti-EBA175 sera in IC were normalized to 100. MFI values for staining of merozoites with anti-EBA175 sera under other conditions are reported relative to MFI for staining of merozoites in IC.

BA: BAPTA-AM

N = 3 independent experiments
