# Supplementary material for: Distinct External Signals Trigger Sequential Release of Apical Organelles during Erythrocyte Invasion by Malaria Parasites
Source: PLoS Pathog. 2010 Feb 5;6(2):e1000746. doi: 10.1371/journal.ppat.1000746 (PMC2816683; doi:10.1371/journal.ppat.1000746)
Supplement: Table S3 — Translocation of rhoptry protein CLAG3.1 to surface of P. falciparum 3D7 and 3D7Δ175 merozoites in response to binding with glyA and RBC ghosts. (0.04 MB DOC) [file ppat.1000746.s013.doc]

| Treatment | Relative MFI for Surface Expression of CLAG3.1(Avg + SD)N=3 | |
| --- | --- | --- |
|  | 3D7 | 3D7Δ175 |
| **IC** | 100 | 100 |
| **EC** | 108.0 ± 2.1 | 113.8 ± 8.7 |
| **EC+GlyA** | 322.2 ± 30.5 | 114.9 ± 8.3 |
| **EC+RBC ghosts** | ND | 289.8 ± 13.4 |

Supplementary Table 3. Translocation of rhoptry protein CLAG3.1 to surface of *P. falciparum* 3D7 and 3D7Δ175 merozoites in response to binding with glyA and RBC ghosts.

MFI: Mean Fluorescence Intensity

^*^MFI values for staining of merozoites with antibodies against CLAG3.1 in IC were normalized to 100. MFI values for staining of merozoites with antibodies against CLAG3.1 under other conditions are reported relative to MFI for staining of merozoites in IC.

N = 3 independent experiments
